# “I don’t think people are ready to trust these algorithms at face value”: trust and the use of machine learning algorithms in the diagnosis of rare disease

**DOI:** 10.1186/s12910-022-00842-4

**Published:** 2022-11-16

**Authors:** Nina Hallowell, Shirlene Badger, Aurelia Sauerbrei, Christoffer Nellåker, Angeliki Kerasidou

**Affiliations:** 1 The Ethox Centre and Wellcome Centre for Ethics and Humanities, Nuffield Department of Population Health, and Big Data Institute, University of Oxford, Oxford, UK; 2 Ilumina, Cambridge, UK; 3 Nuffield Department of Women’s and Reproductive Health and Big Data Institute, University of Oxford, Oxford, UK

**Keywords:** AI, Epistemic and relational trust, Trustworthiness, Computational phenotyping, Electronic phenotyping, Digital phenotyping, Rare diseases, Qualitative research

## Abstract

**Background:**

As the use of AI becomes more pervasive, and computerised systems are used in clinical decision-making, the role of trust in, and the trustworthiness of, AI tools will need to be addressed. Using the case of computational phenotyping to support the diagnosis of rare disease in dysmorphology, this paper explores under what conditions we could place trust in medical AI tools, which employ machine learning.

**Methods:**

Semi-structured qualitative interviews (n = 20) with stakeholders (clinical geneticists, data scientists, bioinformaticians, industry and patient support group spokespersons) who design and/or work with computational phenotyping (CP) systems. The method of constant comparison was used to analyse the interview data.

**Results:**

Interviewees emphasized the importance of establishing trust in the use of CP technology in identifying rare diseases. Trust was formulated in two interrelated ways in these data. First, interviewees talked about the importance of using CP tools within the context of a trust relationship; arguing that patients will need to trust clinicians who use AI tools and that clinicians will need to trust AI developers, if they are to adopt this technology. Second, they described a need to establish trust in the technology itself, or in the knowledge it provides—epistemic trust. Interviewees suggested CP tools used for the diagnosis of rare diseases might be perceived as more trustworthy if the user is able to vouchsafe for the technology’s reliability and accuracy and the person using/developing them is trusted.

**Conclusion:**

This study suggests we need to take deliberate and meticulous steps to design reliable or confidence-worthy AI systems for use in healthcare. In addition, we need to devise reliable or confidence-worthy processes that would give rise to reliable systems; these could take the form of RCTs and/or systems of accountability transparency and responsibility that would signify the epistemic trustworthiness of these tools. words 294.

## Introduction

### Ways of looking at trust

Trust is a relational concept—a disposition or intentional attitude—which is associated with situations of uncertainty, relations of dependency and expectations about future behaviour/intentions [1]. If trust is regarded as a feature of relationships, such that A (truster) trusts B (trusted) to X [2, 3], then trust is A’s attitude regarding B and trustworthiness is an aspect of B namely, “…the commitments, virtues, traits or features [of B] that ground justified or well-placed trust” [4: 24]. Consequently, if B is perceived as, for example: dishonest, incapable of keeping a confidence and performing inconsistently and incompetently, then they will be deemed untrustworthy, as someone that one would not enter into a trust relationship with.

Many authors have observed that trust and trustworthiness are intimately related to notions of risk and uncertainty. According to Bauer [5], trust is “..a belief formed as a result of probabilistic reasoning…a probability that quantifies a belief that the trusted person will in fact do what one is expecting her to do.” [p4], namely, A trusts B because she believes (there is a high probability) that B will X. Likewise, Starke et al. [3] observe that trust is necessary to deal with uncertainty. They point out that in addition to the uncertainty about whether B will X or not X, trust comes into play when A occupies an uncertain or vulnerable relationship with B; A is dependent upon B’s good will to X [2].

Starke et al. [3] argue that trust and trustworthiness can be seen as context dependent; whether A decides to place their trust in B will depend upon A’s willingness to trust, A’s perceptions of B, A’s prior experiences of B and similar situations and the wider context of the task, all of which will speak to B’s trustworthiness and A’s propensity to trust B in this moment. This suggests that the act of trusting is prone to fluctuation and is contingent and conditional. This generates a problem for those who seek to place their trust, for as O’Neill recently put it: “Our aim—everybody’s aim—surely is to trust the trustworthy, but not the untrustworthy.” [6: 293]. The question, she notes, is how do we determine who is trustworthy or where/to whom we should we direct our trust?

### Trust in healthcare

Clinician–Patient relationships can be seen as archetypal examples of trust relationships; insofar as they involve a degree of uncertainty—about prognosis, diagnosis and treatment of disease—dependency relations between the patient and their clinician—the patient is in a vulnerable and uncertain position—and expectations that the participants will behave in proscribed ways, primarily that clinicians will act in patients’ best interests. Clinical relationships involve the sharing of thoughts and feelings and are grounded on the assumption that shared confidences will remain confidential and are based upon the assumption that both participants are trustworthy.

Clinical relationships, like other types of trust relationships, are not static, but are continuously negotiated [7]. Research suggests that not only is continuity of care perceived as important for the development of ongoing perceptions of general practitioners’ (GPs’) trustworthiness [7], but also that a range of relationship or interactional variables such as: information-sharing, respecting patients’ views and values and making patients feel they are respected and taken seriously are regarded as essential to maintaining trusting relationships [1, 8]. Robb and Greenhalgh [9] further note, that in addition to being perceived as highly competent, trustworthy clinicians are seen as empathic, caring and respectful. Indeed, Ward [7] notes, that patients’ expressed lack of trust in locum GPs was put down to GPs’ lack of familiarity with individual patients, a lack of social knowledge and their poor interpersonal skills; this lack of trust led to patients failing to follow the GPs’ advice, being less confident about their diagnosis and failing to taking prescribed medicines or even cashing their prescriptions.

### Ways of looking at trust in AI (in healthcare)

In recent years it has been argued that AI tools, particularly those involving machine learning algorithms, are well placed to undertake some types of healthcare tasks, for example, they can reliably and accurately interpret images in pathology and radiology, thus freeing up clinicians’ time to engage in other aspects of care [10]. Many authors have suggested that as the use of AI becomes more pervasive, and opaque, black box computerised systems are used in clinical decision-making, the role of trust in, and the trustworthiness of, AI tools will need to be addressed [3, 11, 12].

It has been argued that while persons may rely on AI [13] they cannot enter into a trust relationship with it [14] as trust relationships are based on the recognition of another’s good will towards oneself and therefore, only human actors can be involved in trust relationships because “…one can only trust things that have wills” [15: 14). At this point in the paper we do not wish to get into the argument about whether inanimate objects, such as AI tools, can participate in trust relationships (see [15]), nor indeed, whether non-human agents/actors (i.e. technologies/institutions/practices *et cetera*) can be perceived as trustworthy (but see [16]). While we share Metzinger’s [14] worry that designating technology as trustworthy/untrustworthy is a category mistake, we take Starke *et al.’s *[3] that in ordinary language we often describe ourselves as placing (or not) our trust in inanimate objects/institutions and frequently describe them as (un)trustworthy. We also accept that in many of these cases trust relationships are indirect, so that while we may describe an inanimate object, for example, a bridge [3], as trustworthy, and say that we trust it will bear our weight so that we can travel from A to B, we are indirectly placing our trust in the bridge’s designers or builders, who we trust to have endowed it with what we perceive as trustworthy features. What we are concerned with in this paper is not, as Starke et al. point out, whether AI is the sort of thing that can be trusted or perceived as (un) trustworthy but rather under what conditions we should place trust in medical AI [3].

### Empirical studies of trust in AI (in healthcare)

As the above section suggests although there has been much discussion about the need for, and how to conceptualise, trust in AI, there is little empirical research reported about trust in AI tools developed for healthcare practice, particularly the views of AI developers and clinical users. Arguably, it is important to capture the views of these groups as they are involved in designing and/or using AI tools. From the developer/researcher perspective, it is useful to determine how important they think trust is and how they think trustworthiness is/could manifest in the tools they design. From the clinician perspective, as they (will) use these tools in the context of a pre-existing trust relationship, it is important to determine how they think this trust relationship may be impacted by the use of AI tools and also how they characterise the trustworthiness of AI tools.

A recent survey of physicians used a clinical scenario of a hypothetical machine learning risk calculator for predicting the risk of pulmonary embolism to assess the association between physician understanding of algorithmic output, their ability to explain the output to patients and their intended behaviour [17]. The latter was seen as a proxy for trust in the algorithmic output. The hypothetical scenario Diprose et al. [17] used provided a risk estimate (presented in different ways) plus associated treatment recommendations—discharge patient versus refer patient for angiogram. The results indicated that levels of understanding and the explainability of the output were associated with an intention to follow the algorithm’s recommendation. The authors conclude that understanding model output and being able to explain it to patients is associated to greater levels of physician trust, as demonstrated by higher levels of intention to follow the model’s recommendations.

While there are a number of problems with this study, two need to be highlighted. First, the scenario contained limited clinical information about the individual case specifically to prevent the clinicians disagreeing with the ML output thus, prompting them to agree with and, therefore, opt to follow the recommendation. More importantly, the lack of clinical information provided about the case suggests the scenario does not mirror real world diagnostic encounters in which these tools are likely to be deployed. Second, it is unclear that (hypothetical) intended behaviour—following/not following the algorithm’s recommendation—is a proxy for trust, given that it is possible to opt to follow the algorithm’s recommendations, even if one thinks the AI tools are untrustworthy, particularly in forced choice hypothetical situations.

A recent qualitative study undertaken in France [18], which claims to be the first to explore AI researchers’ and clinicians ‘ and other stakeholders’, (e.g ethicists, lawyers) understandings of AI and their implications for healthcare practice, found that a lack of explainability and understanding of AI was perceived as negatively impacting the doctor-patient relationship and potentially interfering with the organisation of healthcare. However, Lai et al. [18] also note that clinicians were positive about the promise of AI suggesting that the use of this technology could improve patient care, but only if the healthcare community is trained to use it and understands how it works. Likewise, they found researchers were positive about the use of this technology in healthcare settings, but this group stressed that AI tools should be used in an assistive rather than a replacement capacity at the present time. Other stakeholders in their study stressed the importance of engaging with and informing patients about the harms and benefits of AI and the need to develop regulatory practices to increase trust in AI. Finally, Lai et al.’s study highlights how different groups of stakeholders need to cooperate and collaborate if AI is to be successfully implemented within the clinic; researchers need to access data from clinicians and require feedback on usability and implementation, and clinicians need insight into how AI is designed and what is a feasible use.

Although these two studies [17, 18] are informative about stakeholder views of the use AI in healthcare, the problem with them is that they use hypothetical scenarios or AI tools are discussed in a generic sense with people who may be less familiar with the technology, although Lai et al. [18] did include a small group of radiologists who were more familiar with these tools.

Hui et al. [19] partially address the latter criticism by exploring patients’ and clinicians’ views of trust in the capacity of the internet of things to support self-management of asthma. Semi-structured interviews were carried out with patients with asthma, some of whom already employ digital devices for asthma management and primary, secondary and community clinicians caring for this patient group. Both groups were asked to design an AI system that could help patients to manage their asthma This study found that while patients accepted and said they would use hypothetical AI tools and that they would be an helpful aid to self-management, they were less confident that AI tools should operate autonomously in certain areas; observing that new management advice/diagnoses should be authorised by clinicians. Clinicians, in turn, observed that while future AI systems could aid self-management, for example, by uploading patient data for clinician review, they were sceptical that these tools would facilitate behaviour change. Like the patients, clinicians also reported that, at present, AI-generated advice and diagnoses require clinician oversight, adding that they would require access to the evidence base before they were prepared to trust system output.

Finally, a recent study of researchers and clinicians actively involved in the development of AI tools, explored their views on how AI algorithms come to be trusted in clinical decision-making [20]. Winter and Carusi [20] argue that accepted components of trust in AI, including: algorithmic explainability and transparency and the ability to justify algorithmic output, are not sufficient to generate trust, but rather, building trust in AI requires negotiation and collaboration between developers and clinical users during the validation process. Focussing on seven interviews with clinicians and AI researchers involved in developing three AI tools (screening, imaging and biomarker algorithms) for early detection of pulmonary hypertension, the authors suggest that the process of validating AI tools is essential for the development of trust in the tool itself. They argue that involving clinical users alongside developers (researchers) in the design, build, and implementation of these tools is fundamental to the process of validation and the building of trust in the tools. Echoing Lai et al. [18], Winter and Carusi see the building of trustworthy AI tools as involving collaboration between different stakeholders, specifically during the validation process. To put it simply, they see epistemic trust in AI tools as grounded upon trust relationships between those who design, build, test and use AI systems, and these trust relationships are built during collaborative practices of AI development. This paper seeks to extend the empirical literature on trust in healthcare AI by exploring the views of those who design and work with computational phenotyping algorithms.

### Computational phenotyping of rare disease

Computational phenotyping (CP) is used to refine the diagnosis of rare diseases that are associated with dysmorphic facial features [21, 22]. Facial photographs (and other biomedical data) of people with a clinical or molecular diagnosis of a rare (usually genetic) disease are used to train facial recognition (FR) algorithms to identify (and classify) the facial phenotypes associated with different rare disorders. Training FR algorithms requires access to digitized photographs of patients who have been diagnosed with different rare diseases and, because these disorders are rare, the development of phenotyping algorithms requires access to datasets from across the world. The Minerva Initiative is an international consortium of commercial, clinical and academic researchers who work on, or support, computational phenotyping using FR algorithms [23]. The consortium was set up to facilitate research in this area by constructing a secure platform for the sharing of digitized facial images and associated data. The aim of the *Facing Ethics Project* was to investigate consortium members’ views of the ethical issues emerging from the use of CP technology in rare disease research and healthcare.

## Methods

Given so little is known about the opinions and values of different stakeholders involved in the development and use of AI tools, this exploratory study used qualitative methods—in-depth interviews. This enabled us to interrogate participants’ understandings of what they perceived as the ethical issues arising from the use of AI technology, specifically CP tools, in healthcare.

### Recruitment of participants

Following receipt of ethical approval from the University of Oxford’s OXTREC research ethics committee, interview participants were recruited by email from the Minerva Consortium’s membership list and by snowballing authors’ and interviewees’ contacts for Industry and Patient support group spokespersons. Forty-seven individuals were invited to participate: two refused, one returned some comments by email, 20 (42%) were consented and the remainder failed to respond despite email follow-up. The final sample of interviewees can be seen as an opportunity sample, which primarily comprised members of the Minerva Consortium plus an industry representative and a patient support group spokesperson (see, Table 1).

**Table 1:** Participant characteristics

*Location*	
Africa	1
Europe	5
Australia	5
US	6
UK	3
Expertise*	
Clinical genetics	9
Paediatric genetics	2
Bioinformatics/data science/computational biology	5
Other clinical speciality	1
Other academic discipline	2
Commercial	2

*N = > 20 as some interviewees fall into two categories for example Academics who are involved in commercial spinouts of CP tools

### Data collection

The data were collected by SB during in-depth interviews (≤ 60 min duration), which took place remotely (telephone/ Skype) during March and April 2019. All the interviews were digitally (voice) recorded with interviewees’ consent. Interviewees’ role in relation to CP and their understanding of the functioning and use of AI systems in healthcare was initially investigated and then they were asked to reflect upon the strengths and weakness of computational phenotyping. Specific questions generated by the interviewees’ responses to these open-ended question and/or based on themes identified in the AI and ethics literature then followed, these were designed to elicit views on: the impact of the use of CP in rare disease research and healthcare, the use of facial images versus other types of personal data, privacy and consent, issues around data-sharing, particularly the difference between public and private initiatives in this area, and the impact of data siloing, algorithmic bias and incidental findings [24].

### Data analysis

The interviews were transcribed and then repeatedly read through by NH to enable the identification of recurrent themes within and between participants’ accounts. The method of constant comparison [25] was used to develop a coding scheme, which was agreed with SB (the interviewer) and then systematically applied to all transcripts. This generated four main categories or themes: *The impact of computational phenotyping on the practice of dysmorphology*, *managing expectations about AI technology*, *trust in AI technology and costs and benefits of using CP tools for diagnosis in dysmorphology*. While trust in AI emerged as a standalone theme in the dataset, it also cut across other themes, for example, participants spoke of the costs and benefits of this technology being realised in a context of trusting relationships and likewise, how expectations of this technology reflect a surfeit or absence of trust. Our data suggested that trust is perceived as foundational to the acceptance of machine learning algorithms in healthcare research and clinical practice, and how this may be achieved is the focus of the analysis presented below.

## Findings

All interviewees talked about the importance of establishing trust in the use of CP technology in rare diseases. Trust was formulated in two interrelated ways within these data. In the first, interviewees talked about the importance of using these tools within the context of a trust relationship; arguing that patients will need to trust clinicians who use AI tools and that clinicians, and patients to a lesser extent, will need to trust AI developers, if they are to adopt this technology. In the second, they described a need to establish trust in the technology itself, or in the knowledge it provides—epistemic trust.

### Placing one’s trust in users and developers

First, interviewees considered the role these tools play in clinical encounters, commenting that clinicians may have different levels of experience in dealing with specific rare disorders, and therefore, using objective CP tools may boost less-experienced clinicians’ diagnostic skills. As P007 reflected, using CP may reduce diagnostic uncertainty in non-specialists by providing them with more objective, standardised diagnoses:

*P007 …the rest [non specialist clinicians] of us could benefit from a little science, I think. So I think that the facial phenotyping software and the development of artificial intelligence that the computer can help you think, “Go this way,” I think is very useful. And I think that it takes out both the individual emphasis that a person puts on, like look at those eyes versus look at that nose, as well as you just standardise the measurements, it is what it is.*

In most instances, the diagnosis of a rare disease involves clinicians drawing upon a range of phenotypic (and genotypic) features in addition to the presence of specific facial features, therefore, CP tools can only provide a small part of the evidentiary basis required. Consequently, the majority of interviewees saw AI technology as a useful adjunct to diagnosis, as providing evidence that can inform, but will not replace, clinical decision-making. P015, a data scientist, reflected that clinicians might come to regard CP tools as useful, if they produce results that agree with them, but may ignore them or come to distrust them, if the results they produce are in disagreement with their assessment.

P015: *I don’t think people [clinicians] are ready to trust these algorithms at face value. I think if it supports what they are saying then that makes sense, it’s in line with other evidence that we have, I think that’s also OK. Oh, we had a mutation in that gene and then the face algorithm is saying that it’s also that gene – that makes sense. But the interesting situation comes when the face algorithm says, no, it’s something completely different. And that, I think, would challenge clinicians, I think, when the face disagrees.*

When talking about trusting the users of CP tools, the interviewees commented at length about how they thought patients would react to their clinicians using this technology for diagnostic purposes. Many observed that scientific accuracy or objectivity of CP technology alone is not enough to foster patients’ trust in machine-led diagnosis, for all diagnoses need interpretation, explanation and justification and that, according to our interviewees, will require clinician input to inspire patients’ trust in the diagnostic process.

*P014: I think a lot of people [patients] always trust the doctor more. And that, you know, let’s say the machine learning goes to a point where someone can have their facial phenotypes analysed by a machine, by artificial intelligence, I think, people always, in my opinion, are going to trust the person’s [clinician’s] opinion more than artificial intelligence, which is probably a good thing. … Because I think you will always have the person, if facial phenotyping at least always has a validation point at the end where it’s done by a specialist.*

Some interviewees observed that patients are wary of machines taking over the responsibility for diagnosis and sought to reassure the interviewer that in their opinion such scenarios are unlikely.

*P019: I think it’s mainly that people [patients] are afraid of a computer taking it over… I think people should be aware that this is in addition to, it’s not going to replace the physician, never going to replace your consultation. I think that’s what people are afraid for, is that they go to the hospital and will go to a machine and they’ll push in some buttons and say, “That’s serious,” or, “You have this and this disease.” … I think that’s the point to make clear, that this is there to help the clinician.*

Indeed, for many of our interviewees the primary healthcare relationship is that which exists between the doctor and their patient, and they were of the view that this trust relationship will not be substituted by the use of AI tools, no matter how accurate and well developed they become, as P010 reflected:

*P010: I don’t want to blame the technology, because the technology is just the tool. It has always been just the tool. For me, it’s all about what use you make of the technology. So it’s all about the person…I believe that human to human interaction is a cornerstone of trust. So this is where I think AI will always come short. . but there are moments, and we need to define what these moments are, where I believe human interaction is what makes us human. So what makes a difference from dealing with a person like a number or dealing with a person like a person.*

In summary, our interviewees were very keen to stress that CP technology is not a standalone technology, but should be used by trusted healthcare professionals to augment their diagnostic skills. They suggested that human intervention was needed in order to inspire patients’ trust in algorithmic output, not least because healthcare requires more than just assigning diagnostic labels, as P010 commented, caring for someone requires that we “…deal with a person like a person” and this is not possible for a machine. Thus, interviewees suggested that CP tools should be used within the context of a pre-existing clinician–patient relationship not least because diagnosis is only a small part of medical care.

Although our interviewees saw relational trust between clinicians and their patients as a condition for the use of CP tools, they acknowledged that both clinicians’ and patients’ trust in this type of technology could be challenged by the involvement of commercial companies in its development. Many of the clinicians commented that there is a need for commercial investment in healthcare in general, and in AI in particular, otherwise some healthcare technologies would not be developed.

*P004: I think we have to be mature about this and think about how commercial partnerships are entered into and engaged in. I mean many good things have happened with public [private] partnerships or that have flowed from commercial operations. I think we just need to acknowledge the real opportunities and having a way of managing that.*

However, some interviewees were less supportive of commercial involvement and speculated that it may undermine the trust that lies at the heart of the clinician–patient relationship. P006 worried that commercial involvement may lead the families they care for to think that their advice was compromised or conflicted:

*I’m sort of nervous of it. I think [patients’] families are not quite at that level yet. So I don’t tend to get too involved with commercial organisations, simply because, when you are speaking to families, you then have to declare an interest, and I think that does impinge on trust a little bit. … I suppose I’m very used to people just feeling that my agenda is their child. And that’s how I want them to feel. I don’t want them to think my agenda is a company that I am working with or something…you just have to be cleaner than clean*.

Many interviewees said they were distrustful of commercial motives and behaviour particularly when it comes to designing AI for use in healthcare and questioned whether this would undermine the development of AI going forward:

*P008: The other issue is, if this were theoretically possible, what I’ve just described, then I simply wouldn’t have enough faith in the big tech companies these days to not think that it wasn’t going to go awry. And that would fundamentally undermine the whole point of the project. Because, if you didn’t have trust, then people [clinicians] wouldn’t do it. And if people wouldn’t do it, the whole thing is pointless.*

A number of interviewees referred to recent scandals such as the unconsented use of data by Cambridge Analytica and Google DeepMind, and talked about the lack of transparency regarding who is curating and protecting patient data, suggesting that there exists a crisis of relational trust between commercial entities who own/handle (health)data and the rest of us, P008 went on to say:

*P008: I think it’s very difficult because you’ve got a handful of very, very large companies which seem to have a lot of control over this world… they are so significantly ahead of the curve in terms of what they are doing, and they are so opaque in terms of what they are doing, that I can’t see people really having genuine trust for a very, very, very long time, and it would only come with massive changes in the way they operated. And I think they get away with it because they have made things which are just so useful and helpful that we just can’t be bothered to do without them. If I fundamentally distrusted Facebook I would have cancelled my account, and I haven’t. So I think there is distrust and dissatisfaction and grumbling, but nevertheless people continue to use what’s on offer. So even in the context of lack of trust, they’ll still exist and they’ll still keep going.*

In other words, in situations where people have no other options to gain a service i.e. if the cost of not ‘trusting’ is to be excluded from a desired good or service, then they may behave “as if” they trust because they are locked into a dependency relationship. Arguably, this does not indicate the existence of a fully-fledged trust relationship, for as P008 notes, there is “distrust or a lack of trust here”, but rather the lack of viable or reasonable alternatives produces what appears to be trust-like behaviour.

Others acknowledged that a lack of trust in commercial developers could be overcome, or mitigated, if the technology they produced was seen to benefit society more widely. They argued that commercial companies should engage in benefit sharing, particularly when AI technology is developed using publically sourced healthcare data.

*P006: Not really. I think it is important that the NHS or whatever healthcare entity, like the third world, if they’re getting involved with that, they see some benefit. If they are giving data that’s going to be used for commercial purposes then I think they should benefit in some ways from that. I think the NHS should benefit from IP that’s generated.*

In summary, many acknowledged that public-private partnerships might have an ever-growing role to play in technology development particularly in publically funded healthcare systems like the National Health Service in the UK. However, many regarded big technology companies as untrustworthy and noted that public benefit sharing may provide a way to inspire public trust in their activities. The interviewees also commented that we not only need companies to behave in a trustworthy way when it comes to using our data, but we also need assurance that the technology they develop is trustworthy. As P003 observed, we need to be reassured that AI tools are subject to stringent reliability and quality assurance checks and that good data governance systems are in place.

*P003: The NHS is having lots of funding problems and I think taking commercial stuff out of all of it is closing the door to so much available resources and money. So I think it’s sensible that we explore all of these options, but I think it’s important that we do our due diligence and we trust that they – that the tests they are doing are accurate, that they are reproducible, that the quality is high enough, that they look after the data properly, they analyse the data fully, you know, all of those sorts of things.*

### Trusting technology: epistemic trust in AI

While it is important to trust those who use and/or develop AI technology our interviewees also commented that clinicians and patients need to trust the technology itself, or the knowledge it produces. A number of interviewees observed that it is difficult for people in general to place trust in CP technology at this point in time because it is novel.

*P011: And will people trust those results? They probably will. I think it’s just people have a trouble trusting something that’s new, but once it becomes part of like, …I think it’s that people find these new technologies a bit scary at first but they just become normal. So [CP technology] will become just what everybody gets used to using.*

Epistemic trust in CP tools was seen as a future development, and many interviewees observed that trust in algorithmic output could be built up through the collection of evidence that these tools work and that the knowledge they produce is trustworthy.

*P007: Well, I think it’s proof, accruing evidence is what will lead to trust. I think there’s certainly potential for harm, in that it really depends on who is programming the machine, what it’s being told to do… I don’t think it’s appropriate to have [a ton of] trust in machine learning yet. I don’t know that it’s proven its point and worth completely.*

According to interviewees, clinicians’ current lack of epistemic trust in CP tools arises because of the novelty of the technology and from their lack of experience in using them for diagnostic purposes. As P006 observed, it is possible that CP tools, like other medical tools, will come to be seen as providing clinicians with reliable and accurate knowledge given time:

*P006: I think the trust is really in the scientific method and actually saying, well, what are you actually trying to do? …So I think it’s just a matter of working out what you want a machine to do and how are you going to train it to do that and how are you then going to monitor its performance. And it’s the same as any piece of machinery that you’d use in the clinic, you’ve got to have ways of monitoring how it’s performing….So trust, to me, is really, I mean do I trust my stethoscope? Yes, because I’ve been using it for nearly 40 years. So do I trust an ECG? Yes, I do, if it’s done by someone who is experienced and knows where to place the leads and knows how to calm the child down. …. And the precautionary principle is, don’t do anything in medicine unless you know what you’re doing, and you don’t trust things you don’t know. And have a reasonable idea, if you’ve got a machine there, have a reasonable idea how it’s working.*

Interviewees suggested that as far as clinicians are concerned, developing trust in any technology is reliant on one’s experience of using it; trust is learnt. As P006 commented: “…*you gain trust through experience. You can’t demand trust, you can’t demand that somebody trusts a computer.”* But technology itself also earns our trust, insofar as it comes to be perceived as trustworthy because it is validated by other types of evidence that are used to calibrate the accuracy of its performance. As P015 commented new technologies are often introduced into the clinic alongside those they will replace, with the result that epistemic trust is earned or built up over time.

*Trust, yeah you have to gain it. You can’t just put a computer in a room and say, “Right, you need to trust it now it’s going to diagnose all your patients based on a face,” that’s not the way it works. I mean many of these clinicians have had very many years of experience as well, so they’ve seen a lot, so they think they also are right (laughs)…Yeah, the stereotypical way of getting a new technology into diagnostics is that you run it in parallel for a time, and then sometimes it agrees with the existing technology that provides results and then sometimes it gives new insights and then eventually the trust is there and you make the switch. … People have to be willing to take that leap of faith to run it in parallel for a while.*

With regard to CP tools, interviewees also observed that molecular testing can be used as a backup or failsafe for knowledge produced by these AI tools and vice versa. In other words, clinicians’ perceptions of trustworthiness, at least in the short-term, will be based on some form of external (objective) validation.

*P002: To be honest, at this moment, it’s [CP technology] not good enough. But I can say it’s not good because I have the experience. But in 10–15 years’ time, [when] people like me [specialist dysmorphologists] are not working any more if you blindly trust the outcome of the algorithm and stop thinking then you might make the wrong diagnosis. But if you use it, if you can actually still confirm by molecular testing the outcome of the algorithm, then it’s helpful….*

This observation led some to question whether CP technology would ever be perceived as trustworthy enough to operate without a human in the loop determining the accuracy or trustworthiness of algorithmic output. This group argued that human oversight remains essential for the generation of epistemic trust in AI. Accordingly, the majority of interviewees speculated that patients would be likely to view relational and epistemic trust as existing in an interdependent relationship, one in which epistemic trust in CP tools is seen as dependent upon relational trust in their clinical users. In other words, as far as our interviewees were concerned if a trusted person—your doctor—uses AI, then you are more likely to trust the algorithmic output.

*P011: I think most people think that the algorithms are better, I would have thought. I think they are very good, all of the algorithms. But I think, at the end of the day, there is a doctor involved and … I think people are more likely to trust something where the doctor is involved, rather than just, “This algorithm said you’ve got this diagnosis.” Whereas if the algorithm is helping the doctor make a decision or helping a lab find the variant, I think that’s different. I think helps people have trust.*

Our data suggest that clinicians’ perceptions of the trustworthiness of CP tools will be related to perceptions of their reliability and validity, seeing that they work correctly. Indeed, the idea that AI technology should undergo rigorous reliability testing and cross-validation recurred throughout these interviews:

*P008: So the AI itself, I think that trust would be established by illustrating that it worked, much like I would trust a new cardiovascular drug if there was a good RCT showing that it worked. I don’t think I’d need any more evidence. That just good, high quality level of evidence would do the trick for me.*

Many commented that epistemic trust must be built upon transparency about, and awareness of, how algorithms work, rather than having “blind faith “in algorithmic output. One of the data scientists we interviewed described how they were developing interpretable CP algorithms for clinicians, algorithms that are more transparent and will list the components on which their decisions are based. In contrast, others suggested that the development of explainable CP tools was a long way off and is complicated by the fact that current CP-based diagnostics are probabilistic and are not based on anatomical features like human diagnosis. In other words, algorithmic diagnoses are presented in an unfamiliar format.

*P015: The computer analyses this face and it says, yes, they are the same. But it cannot say in a concrete way it’s the nose or it’s –, it’s like it’s generalising and it’s seeing something. And that’s then very hard to explain to clinicians, because clinicians are used to looking at a face and saying, oh, it’s the ears, or it’s the eyes. So translating, I think that’s a problem with some of the deep learning methods, they are abstract and that makes it very hard for a clinician to understand why the computer is saying what it’s saying. Because ultimately the computer just pumps out a number it says, yeah, there is a probability of 83.2% that this person has something or other.*

Others raised the issue of algorithmic bias particularly, its impact on different ethnic groups, suggesting that the use of biased algorithms could lead to users questioning the trustworthiness of CP tools.

*P003: So I think really what you’re asking is how will people have faith and trust that the algorithms are not biased and they are accurate? I guess by becoming more prevalent and proving that they work, is the obvious answer. That if they make diagnoses that are then confirmed by a molecular diagnosis, then that is proof in itself. I haven’t used this software so I can’t speak from personal experience, but I think the concern will always be, with rare diseases, has there been enough data?*

Bias is a problem in CP research, as the lack of numbers of people with rare diseases always leads to questions concerning representativeness of datasets used in algorithm training and, consequently, the reliability and validity of algorithmic output. As one of the data scientists reflected:

*P015: So I think, yeah, I think the biggest thing is that the methods need to be able to handle not having 50 patients. It’s possible but it’s, yeah, it’s tough. You know, you’re on the border of your power and that means that your confidence goes down a bit, which then makes it harder to accept what the computer is saying.*

Greater transparency about the composition of data training sets and how algorithms work was seen as offering, at least, a partial solution to this problem, as P020, an industry spokesperson said:

*Well, the first thing that you need to do is you need to be transparent. So you need to publish your work… In many senses it’s easier to trust artificial intelligence if you have more information, right. So as long as you can make sure that it has seen enough cases, enough diverse cases and the network is up and running…So I think trust is going to get a different perspective in the future.*

Finally, a small group considered that failing to understand how AI works is not necessarily a barrier to using it. Indeed, P012 observed that the need for transparent and explainable AI may have been over-emphasised by commentators, not least because understanding how some clinicians have arrived at a particular diagnosis is not always clear, and that less-experienced clinicians are often unable to justify their decisions.

*P012: I mean it’s definitely challenging to understand more about what these algorithms do. They are often referred to as black boxes, but still I think many clinical experts are black boxes too, all their trainees too, right, and they can hardly explain why they come up with a certain differential diagnosis. So it’s not that different, I think. Ultimately you have to trust in the technology, in the knowledge or service it provides.*

Our data suggest that having trust in CP tools themselves is as important as trusting those who use or develop them. According to the interviewees, epistemic trust in CP tools is conditional and contingent; they observed that clinicians will learn to trust these tools through repeated use and trust is earned on the basis of their performance. In other words, AI tools, like any new technology, come to be perceived as trustworthy because they work well, producing reliable and externally validated results. Moreover, it was clear that greater transparency regarding the ways in which tools work and how they are trained would be important to generate perceptions of trustworthiness.

## Discussion

This study suggests that trust in AI tools deployed in healthcare involves relational trust, trusting those who use and develop AI, and epistemic trust, trusting in the knowledge produced by the tools. Our interviewees suggested CP tools used for the diagnosis of rare diseases might be perceived as more trustworthy, by clinicians and patients alike, if the user is able to vouchsafe for the technology’s reliability and accuracy and the person using/developing them is trusted. In the remainder of this paper we will look in more detail at issues around the relationship between relational and epistemic trust in the use of AI in healthcare.

### Who should we trust?

As many authors have observed [2, 3], trust is grounded within social interaction, and aspects of this social context, the power, expertise/knowledge and expectations of the truster and trusted will affect the promotion of trust within relationships. Trust in clinician–patient relationships similarly derives from personal and also structural factors; it is vested in an individual clinician’s expertise or knowledge [26] as well as trust in the institutions and overall system that supports and regulates the provision of healthcare [27]. Viewed this way, epistemic trust, being able to rely on healthcare professionals’ testimony regarding our health, forms the basis of relational trust, the belief in the expertise and goodwill of the doctors towards the patient. In other words, relational trust is seen as grounded upon epistemic trust. Despite the advent of the internet-informed ‘expert’ patient and AI tools, it still seems reasonable to assume that clinicians have the most (relevant] expertise in this relationship. Our interviewees similarly hypothesised that, from a patient perspective, trust in the use of CP tools will be founded on trusting the clinicians who use these tools; who will be seen as the final arbiters of AI’s trustworthiness. Indeed, this study, like those reported by Lai et al. [18] and Hui et al. [19], suggests that those who work in clinical genetics and rare disease research do not anticipate that CP tools will be used without a human in the loop [10], as all predicted that these tools will be used to augment human decision-making in the context of a pre-existing trust relationship. So to return to the earlier question raised by O’Neil [6] “how do we determine who is trustworthy or where/to whom we should we direct our trust?” It would appear that when it comes to the use of AI tools in healthcare, patients should put their trust in those clinicians who can demonstrate they have the skills and expertise to use these tools.

But while our interviewees regarded future patients as trusting them and/or other healthcare professionals to use AI tools wisely, they were somewhat sceptical of the motives of commercial developers of AI systems, particularly the larger technology companies. A number of the interviewees suggested that AI developers might come to be perceived as more trustworthy by the wider society (and clinicians themselves) if they were to engage in more benefit sharing. However, as Kerasidou [12] has recently observed it is unclear that profit sharing alone will increase clinicians’ and patients’ perceptions of the trustworthiness of technology developers or the technology itself. Indeed, she argues that because trustworthiness is “self-motivated and self-regulated”, we should focus less on nudging commercial AI developers to become more trustworthy and put more emphasis on regulating their behaviour and developing transparent systems of accountability. In other words, we should introduce forms of external regulation that will lead commercial actors to act in more acceptable ways.

### What counts (should count) as trustworthy AI?

However, as our interviewees pointed out, while trusting those who develop and use CP tools is important, one also needs to trust the performance of the tools themselves. In the introduction to this paper we claimed that we were not interested in the question of whether non-human actors—such as AI tools—were the sorts of things that can be designated as trustworthy [14, 15]. Following Starke et al. [3] we suggested that the existence of indirect trust in AI designers and users might be enough to say that we have, or may have, a trusting attitude towards the technology itself.

“In its indirect, weaker sense, trust in AI does not require a fully independent agency of the program itself but rather ties trust to the intentions of its developers or those involved in its quality control, promoting ‘indirect trust in the humans related to the technology’. For example, we may trust a system of medical AI because we trust the people who develop and regulate it. Even in this very limited sense, it may already be plausible to describe a potential attitude towards medical AI as ‘trusting’.” [3:157].

Starke et al. [3] suggest that if we are to regard trust as an essential component of our relationships with AI systems/tools, then we need to adopt a view of trust, which places it in a three dimensional framework in which:

“…the decision to trust an AI- based program depends on features of the trustor (e.g., overall willingness to trust), and the context (e.g., level of risk) in combination with the perceived reliability, competence, and intentions of the program.” [3:158].

According to this model, judging a system’s trustworthiness is dependent on *assessments of its reliability*—whether it works in the same way at different times and in different conditions—*judgements of its competence*—does it produce accurate and valid measurements, i.e., is it measuring what it claims to measure—and finally, *exploring its (indirect) intentions*—namely, the developers’/designers’ conflicts of interest, the system’s transparency and the representativeness of the data used in its training, in other words, explainability and openness is seen as essential to foster trust in the system’s intentions. Starke et al.’s framework posits that trusting in AI requires the truster to engage with these three dimensions, but they note that these three dimensions do not have to be met equally for individuals to perceive the system as trustworthy.

Another way of understanding Starke et al.’s [3] three dimensions of trust in AI is through the lens of epistemic trust [28]. What the authors are describing are features of epistemically reliable tools that operate as they should under the conditions and purposes for which they are employed. What indicates the reliability of AI tools for use in medical practice would be the fact that these tools are designed and rigorously tested to produce reliable claims. As Schwab notes, claims produced by reliable processes are exactly what we look for in medical practice [28]. These sentiments are implicitly echoed in Wang et al.’s review [29], which suggests that medical AI should be evaluated in randomised controlled trials that follow strict reporting protocols.

As the data outlined above demonstrate, issues pertaining to AI’s epistemic features, or to use Starke et al.’s terminology, its three dimensions of trust, were repeatedly mentioned in our study and described as important. The interviewees frequently raised the issue of reliability and competence as they talked about checking that data used in training the tools were representative and that the outputs of CP tools were validated using other types of evidence, including clinical expertise.

Following Starke et al. [3] our interviewees also observed that designing transparent systems and explainable AI is important to increase the perceived trustworthiness of AI systems and argued that one also needs to take into account the motivations and intentions of AI designers and developers. The extent to which the trustworthiness of the developers’ intentions versus the trustworthiness of the intentions designed into the systems themselves (transparency and explainable AI) are important for overall perceptions of a system’s trustworthiness was not clearly articulated by Starke et al., nor by our interviewees. Indeed, it is unclear how the system’s intentions interact with those of its designers, although it was apparent that our interviewees regarded developers’ conflicts of interest as a barrier to perceptions of trustworthiness of AI systems.^1^

But how do increased reliability, competence and the system’s intentions relate to trust? While these characteristics may enable us to overcome some of the uncertainty, which is an integral aspect of trust relationships [3, 15], we can ask if acknowledging the ability to more accurately and reliably predict outcomes is the same as trusting as Starke et al., suggest? In other words, is there a difference between a well-intentioned/reliable/competent and a trustworthy diagnosis? C.Kerasidou et al. [13] maintain that fostering reliance is more appropriate than developing trust in our dealings with commercial AI developers (here we substitute AI technology) because reliance can be supported by strong legal and regulatory frameworks, whereas trust, as an attitudinal relationship, cannot. They note that reliance is about ensuring predictability, in contrast to trust, which is based on perceptions of the trusted’s goodwill towards the truster [2, 15]. They observe that putting in place the regulatory mechanisms to ensure greater reliance in AI technologies will provide the conditions for trust to emerge as an end in itself rather than a means towards an end. In others words, if we can rely on those who design the technology, and/or the technology itself, we may begin to trust them/it.

Graham [30] aims to extend this reliance-based model in a recent paper about developing ethical data-sharing practices, in which he argues that interactions with commercial actors should be based on confidence rather than trust. In contrast to reliance, which, he observes, depends on expectations of predictability, confidence is a form of assured reliance, such that B doing X is not only predictable, but also is assured, i.e. guaranteed. Although Graham fails to clearly articulate the distinction between assured reliance, or confidence, and mere reliance [13], he does outline criteria for developing confidence-worthy systems for data sharing (here we substitute AI systems), arguing that they would involve: meaningful (i.e. understandable) transparency arrangements that can be checked, clear mechanisms of accountability, and assurances that the data involved are representative (i.e. not biased) and have a demonstrable social purpose (i.e. are working for the public good). Graham [30] argues that confidence, unlike trust, is not dependent upon A’s recognition of B’s good will [2, 15], but, like reliance [13], is an enforceable obligation. Consequently, if AI development does not meet the conditions outlined above, for example, if it fails to meet the transparency requirement for confidence, then AI developers will be sanctioned.

Our data suggest that although good intentions, reliability and competence can be seen as conditions for perceiving CP tools as more predictable [13, 30] and therefore, confidence-worthy or reliable, they are not sufficient for perceiving tools as trustworthy, for, as many interviewees pointed out, diagnosis is not just about correctly labelling or categorising patients but also involves treating them as persons or treating them with respect [8]. Thus, while our interviewees saw CP technology as an expert tool (a reliable and competent tool) they did not advocate that these tools should replace trusted human experts.

In summary, the impending adoption of AI tools to support diagnostic decisions in the clinic has led to a discussion about the role and importance of trust [12, 31, 32]. While most would agree that it is essential that patients have trust in clinical users of AI, it has been argued that instead of promoting trust in AI developers or indeed, AI systems, it would be preferable to see these relationships as based on reliance [13] or confidence [30].

Finally, we want to point out that while transparent AI systems may be seen as having more trustworthy intentions than opaque or unexplainable ones, as noted above, increased transparency and explainability alone are not enough to generate trust in the use of AI systems, for as Sand et al. [33] argue, it is important that those who use these AI systems use them responsibly. According to Sand et al., when implementing AI systems in healthcare we need to “focus on forward-looking responsibilities of physicians using such systems.” [33: 163], which involves encouraging the development of a range of competencies and virtues in those who use AI tools, including: understanding and assessing the outputs and inputs of AI tools, reflexive awareness about one’s knowledge and competency in using the tools, monitoring the performance of tools over time and being able to explain uncertainty regarding output to patients, amongst other things. In other words, Sand et al., argue that we cannot see trustworthiness as located within AI (or any other) tools or systems per se, but in the relationship between the tools, the clinician and the patient. As noted above, similar sentiments were expressed by both Lai et al. [18] and Winter and Carusi [20], who observed that ongoing collaborative relationships between AI developers and their clinical users are essential to the development of trustworthy AI tools. In other words, epistemic trust is grounded upon relational trust.

Prior to concluding, it is important to address the limitations of this research. This project only looked at the views of developers and clinicians, primarily members of the Minerva Consortium, who are involved in using or developing CP. Consequently, the data reflect their perceptions of CP tools and their hypotheses concerning how patients/other clinicians will react to them if and when they are deployed in the clinic. The fact that the bulk of interviewees were recruited from the Minerva Consortium, and are involved in either providing data for the development of CP tools or developing these tools, could mean that they have an interest in presenting this technology as potentially trustworthy. On the other hand, one could argue that, at least as far as clinicians in this study were concerned, it was not in their interest to claim that these technologies are trustworthy, or will be perceived as trustworthy enough to undertake diagnosis without human input, as they would be talking themselves out of a job [34]. Bearing this in mind we suggest that future research, should not only explore what clinical users and developers think are the ethical issues arising from the use of CP technology, but also look at those views of patients, whose diagnoses are/will be supported by these tools.

In addition, it must be noted that, despite the consistency of responses across the interviews, the interviewees in this study come from a wide variety of geographical locations (with different healthcare systems and priorities) and areas of expertise, including those who use CP technologies and/or design and build them. Arguably, this diversity can be seen as a strength of this study as it enabled us to explore how a diverse group envisage the issue of trust in relation to CP tools from a variety of perspectives.

Finally, we need to address the question of whether our arguments are only pertinent to the use of AI to diagnose rare diseases? Indeed, given the degree of uncertainty involved in the diagnosis of rare disease, is trust in CP assisted diagnoses more difficult to achieve than in other areas of medicine which deploy AI tools, for example digital pathology or radiology? While it is possible that higher levels of uncertainty in the diagnosis of rare disease and the lack of large datasets for training CP algorithms may influence the conditions needed to support trust in this clinical context, we contend that the findings generated in the present study may be applicable to the use of AI tools in other areas of medicine. Although the empirical literature in this area is sparse, the concerns and opinions expressed by our interviewees echo those of recent studies, which argue that AI should be used in the context of a trusted relationship [18, 19] and that epistemic trust in AI is grounded in collaborative relationships [20].

## Conclusion

Trust is fundamental to the clinician–patient relationship [35], the patient trusts that their clinician will act in their best interests and has requisite expertise to diagnose and treat them effectively. While trust is integral to all diagnostic encounters, it is perhaps tested more severely in the case of rare diseases, not least because on many occasions the clinician’s expertise or knowledge may be challenged or lacking, primarily because rare diseases are, as their name implies, rarely encountered in medical practice. CP tools offer a way to enhance diagnostic yield in rare disease by augmenting clinicians’ diagnostic skills. Our data indicated that clinical users and AI developers perceive a strong link between relational and epistemic trust when it comes to the introduction and use of AI tools in the clinic. They think that AI tools need to be developed in a way that makes them epistemically reliable and acknowledged that perceptions of their reliability will be earned and learnt over time through their use by trusted individuals. In other words, clinicians’ positive experiences of using CP tools will result in greater trust in their diagnostic abilities. In this sense, trust in AI systems is contingent and conditional. It is based upon the existence of processes to test and demonstrate their reliability, and the ongoing positive/appropriate performance of AI systems within a pre-existing clinical relationship. Indeed, it was clear, that our interviewees believed that future patients would most likely be of the opinion that if it is good enough for my clinician to use it, then it is good enough for me, thus, providing the link between relational and epistemic trust in this domain.

In conclusion, this study suggests we need to take deliberate and meticulous steps to design reliable or confidence-worthy AI systems for use in healthcare. In addition, we need to devise reliable or confidence-worthy processes that would give rise to reliable systems; these could take the form of RCTs and/or systems of accountability transparency and responsibility that would signify the epistemic trustworthiness of these tools. Once such systems and processes are in place, AI tools could help to promote the trusting clinician–patient relationship.

## Data Availability

All relevant data are within the manuscript. The data generated and analysed during the current study are not publicly available as participants did not consent to data archiving and data will be destroyed 5 years after the end of the study as per OxTREC requirements. Specific data is available from the first author on request.

## Footnotes

1 Regarding this point, we think it is important to note that untrustworthy AI systems (model behaviours) will not always be the result of designers’/developers’ malicious intent, but may arise from human error: a lack of insight, a lack of diligence in seeking out representative training datasets or omitting to design transparency into the system. Although we accept that these things may be (indirectly) influenced by conflicts of interests, we think it is important to acknowledge that these negative effects could also happen unintentionally unless deliberate and meticulous steps are taken to prevent, quantify and counteract them.
